# Clinical Applications of Optical Coherence Tomography and Optical Coherence Tomography Angiography in Uveal Melanoma: A Narrative Review

**DOI:** 10.3390/diagnostics15192421

**Published:** 2025-09-23

**Authors:** Mario Troisi, Livio Vitiello, Filippo Lixi, Mihaela Madalina Timofte Zorila, Giulia Abbinante, Alfonso Pellegrino, Assem Namazbayeva, Ginevra Giovanna Adamo, Giulia Coco, Alberto Cuccu, Giuseppe Giannaccare

**Affiliations:** 1 Eye Clinic, Department of Neurosciences, Reproductive and Odontostomatological Sciences, University of Naples Federico II, 80131 Naples, Italy; troisi165@gmail.com; 2 Ophthalmologic Unit, University Hospital of Salerno, 84100 Salerno, Italy; 3 Eye Unit, Luigi Curto Hospital, Azienda Sanitaria Locale Salerno, 84035 Polla, Italy; giulia.abbinante@gmail.com (G.A.); al.pellegrino@aslsalerno.it (A.P.); 4 Eye Clinic, Department of Surgical Sciences, University of Cagliari, Via Università 40, 09124 Cagliari, Italy; filippo.lixi@unica.it (F.L.); a.cuccu@aoucagliari.it (A.C.); giuseppe.giannaccare@gmail.com (G.G.); 5 Faculty of Medicine, Grigore T. Popa University of Medicine and Pharmacy, 16 Universitatii Street, 700115 Iasi, Romania; madalinatim@yahoo.com; 6 Department of Ophthalmology, Cai Ferate Clinical Hospital, 1 Garabet Ibraileanu Street, 700506 Iasi, Romania; 7 Kazakh Eye Research Institute, Tole Bi Street 95a, Almaty 050000, Kazakhstan; namazbayeva2020@bk.ru; 8 Department of Translational Medicine, University of Ferrara, Via Ludovico Ariosto 35, 44121 Ferarra, Italy; dmagvr@unife.it; 9 Sant’Anna University Hospital, Via Aldo Moro 8, 44124 Ferrara, Italy; 10 Ophthalmology Unit, Department of Clinical Sciences and Translational Medicine, University of Rome Tor Vergata, Via Montpellier 1, 00133 Rome, Italy; giulia.coco@uniroma2.it

**Keywords:** uveal melanoma, choroidal melanoma, iris melanoma, optical coherence tomography, optical coherence tomography angiography, anterior segment imaging, multimodal imaging, choroidal imaging, ocular oncology, artificial intelligence

## Abstract

Uveal melanoma is the most common primary intraocular malignancy in adults, most frequently arising from the choroid, followed by the ciliary body and iris. Its diagnosis and management require precise characterization of tumor morphology, localization, and associated complications to optimize visual and systemic outcomes. Recent advances in optical coherence tomography (OCT), anterior segment OCT (AS-OCT), and OCT angiography (OCTA) have expanded the ophthalmologist’s ability to non-invasively visualize structural and vascular changes associated with this disease. In fact, enhanced depth imaging (EDI) and swept-source (SS) OCT can provide detailed views of deep ocular structures, enabling early detection of hallmark features such as subretinal fluid, retinal pigment epithelium disruption, and dome- or mushroom-shaped choroidal elevations; AS-OCT improves evaluation of lesions of the anterior segment, revealing iris architecture distortion and angle involvement; OCTA facilitates the visualization of abnormal tumor vasculature and detection of radiation-induced microvascular changes, including capillary dropout and foveal avascular zone enlargement. Moreover, these imaging modalities have demonstrated utility in differentiating uveal melanoma from pseudomelanomas, such as choroidal nevi, hemangiomas, and metastases. The present review aims at objectively assessing the use of OCT and OCTA in the diagnosis, treatment, and follow up of ocular melanoma, emphasizing their crucial role in identifying pathologic biomarkers of this potentially fatal ocular disease.

## 1. Introduction

Uveal melanoma (UM) is the most common primary intraocular malignancy in adults, arising from melanocytes within the uveal tract, which includes the iris, ciliary body, and choroid. Among these, choroidal melanoma represents the vast majority of cases, followed by tumors originating from the ciliary body and, more rarely, the iris [1]. Although the incidence of UM has remained relatively stable over recent decades, its clinical relevance is high due to the significant risk of local visual impairment and systemic metastasis, particularly to the liver. Consequently, early and accurate diagnosis remains essential for preserving vision and optimizing survival prognosis [2].

Traditionally, evaluation and monitoring of UM have relied on clinical examination, including indirect ophthalmoscopy and fundus biomicroscopy, combined with a range of diagnostic modalities such as ultrasonography (US), fluorescein angiography (FA), and indocyanine green angiography (ICGA) [3]. These tools provide crucial information on tumor size, internal reflectivity, and vascular patterns. However, each method presents limitations. For instance, FA and ICGA require intravenous injection of dye, an invasive procedure that may induce adverse reactions such as nausea, vomiting, and, in rare cases, anaphylaxis [4,5]. Moreover, they are often limited in spatial resolution or depth penetration. Similarly, US may not adequately characterize surface alterations or subtle retinal changes. These shortcomings have fostered growing interest in high-resolution, non-invasive imaging modalities for enhanced tumor assessment [3,6].

Optical coherence tomography (OCT) has emerged as a pivotal tool in ocular oncology, offering cross-sectional, micrometer-scale imaging of ocular structures. It enables detailed evaluation of retinal and choroidal anatomy, facilitating the detection of subretinal fluid, retinal pigment epithelium (RPE) irregularities, retinal thinning, and other tumor-induced alterations [7,8]. OCT angiography (OCTA) builds upon these capabilities by visualizing retinal and choroidal vasculature without requiring intravenous contrast agents. Its noninvasiveness, rapid acquisition time, and ability to assess blood flow make it particularly suited for longitudinal monitoring. These advantages position OCTA as a valuable complement to conventional angiographic techniques, especially in the detection of microvascular abnormalities and early complications such as radiation retinopathy [7,9].

Recent studies have emphasized the diagnostic utility of OCT and OCTA in differentiating small melanomas from pseudomelanomas, detecting early vascular disturbances, and informing treatment strategies such as plaque brachytherapy or transpupillary thermotherapy [6,10,11]. In particular, OCTA has demonstrated the capacity to identify parafoveal capillary dropout, choriocapillaris non-perfusion, and irregular tumor vasculature—changes that may precede structural alterations visible on traditional imaging modalities [7,12].

Given the increasing clinical relevance of OCT-based technologies, this narrative review aims at summarizing current evidence on the role of OCT, anterior segment OCT (AS-OCT), and OCTA in the diagnosis, characterization, and management of UM. Special emphasis is placed on their use across the spectrum of uveal tumors, including anterior segment involvement, and their utility in both treatment-naïve and post-therapy settings. A comprehensive overview of how these imaging modalities are reshaping the modern approach to uveal melanoma care will be provided by integrating recent advancements and clinical applications. Because much of the OCT/OCTA literature in UM comprises single-center, retrospective series with non-uniform acquisition/segmentation, we treat reported effect sizes as exploratory; accordingly, we foreground multicenter and longitudinal studies, flag reports with repeatability or inter-device agreement, and specify where standardization is still needed.

## 2. Uveal Melanoma: Bridging Genes, Pathogenesis, and Diagnosis

### 2.1. Epidemiology and Pathogenesis

Although relatively rare, uveal melanoma (UM) exhibits notable geographic and ethnic variability, with reported annual incidence rates of 4 to 7 cases per million in Europe and North America that decrease in Asia and Africa [13]. Unlike cutaneous melanoma, the incidence of UM has remained relatively stable over the past decades [14,15]. The condition predominantly affects Caucasian populations, with a markedly lower incidence among individuals of African or Asian descent—an epidemiological pattern likely related to differences in ocular pigmentation and genetic predisposition [15,16,17].

The disease exhibits a peak incidence between the sixth and seventh decades of life, with a slight male predominance reported in several epidemiological series [18]. Anatomically, the choroid is involved in approximately 85–90% of cases, followed by the ciliary body (5–8%) and iris (3–5%) [19]. Tumors arising from the ciliary body tend to have a worse prognosis due to delayed detection and higher rates of extraocular extension and systemic metastasis at diagnosis [20].

From a pathobiological standpoint, UM is characterized by the malignant transformation of neural crest-derived melanocytes located within the uveal tract [21]. Unlike cutaneous melanoma, UM is not typically associated with ultraviolet (UV) radiation exposure, although light iris color and chronic actinic damage may still confer a certain degree of risk [22,23]. Genetic studies have identified several key driver mutations and chromosomal alterations involved in the pathogenesis of UM. In particular, activating mutations in the *GNAQ* and *GNA11* genes—encoding Gαq and Gα11 proteins involved in the MAPK pathway—are present in over 80% of tumors, constituting early events in melanocytic oncogenesis [24].

Subsequent progression to malignancy is associated with additional alterations, including mutations in *BAP1*, a tumor suppressor gene located on chromosome 3. Loss of *BAP1* expression correlates with monosomy 3 and is strongly associated with metastatic potential and poor survival [25]. Other secondary mutations involve *SF3B1* and *EIF1AX*, which are generally found in tumors with more favorable outcomes [26,27]. Gene expression profiling (GEP) has enabled the molecular stratification of UM into risk categories, with Class 1 tumors associated with low metastatic risk and Class 2 tumors demonstrating aggressive behavior and a high propensity for hepatic dissemination [28].

Tumor dissemination in UM occurs primarily via hematogenous routes, as the uveal tract lacks lymphatic drainage. The liver is the predominant site of metastasis, affected in up to 90% of metastatic cases, often with no curative options once systemic spread has occurred [29].

Altogether, the pathogenesis of UM reflects a complex interplay of genetic, anatomical, and racial factors, reinforcing the importance of early detection and precise molecular characterization for risk stratification, prognosis, and individualized management.

### 2.2. Clinical Presentation and Diagnostic Challenges

The clinical manifestation of uveal melanoma largely depends on the tumor’s size and anatomical location [30]. While many patients report symptoms such as blurred vision, photopsia, or visual field loss, a significant proportion—up to 30%—remain asymptomatic, with lesions identified during routine ophthalmic examinations [30,31]. Visual disturbances can also include ocular discomfort, floaters, metamorphopsia, or increased intraocular pressure [1]. Choroidal melanomas typically appear as dome-shaped or mushroom-like subretinal masses, the latter resulting from rupture through Bruch’s membrane. These lesions may vary in pigmentation, from dark brown to amelanotic, and are sometimes accompanied by exudative retinal detachment, contributing to visual loss [32].

Ciliary body melanomas may go undetected until they exert mass effects, such as lens displacement or angle involvement leading to secondary glaucoma [33]. Sentinel vessels on the sclera or abnormal pupil dilation may offer external clues.

In contrast, iris melanomas are more readily observed due to their anterior location and usually present as enlarging pigmented lesions, most commonly in the inferior iris quadrant [34]. They can lead to localized cataract formation, hyphema, or secondary glaucoma if angle structures are compromised [35]. Rare variants—such as diffuse, ring, or tapioca iris melanomas—can present with unilateral heterochromia, nodular growths, or sustained intraocular pressure elevation [36].

Diagnostic evaluation relies on a comprehensive ocular examination, including slit-lamp biomicroscopy and indirect ophthalmoscopy. Ancillary testing such as B-scan ultrasonography, OCT, and fluorescein angiography further aid in tumor characterization [3,37]. While the clinical accuracy for diagnosing uveal melanoma now exceeds 99%, studies have shown that initial misdiagnosis still occurs in up to 23% of cases, potentially leading to delayed treatment and higher risk of primary enucleation [30]. Recently, OCT and OCTA have become valuable non-invasive tools to visualize retinal and choroidal structures in detail, offering complementary insights in lesion assessment and vascular pattern analysis [10,11].

## 3. Optical Coherence Tomography in Uveal Melanoma

### 3.1. Technical Principles and Modalities

OCT is a non-invasive imaging modality that utilizes low-coherence interferometry with near-infrared light to produce high-resolution, cross-sectional images of ocular tissues [38]. Unlike ultrasound, which relies on acoustic wave reflections, OCT measures light backscatter, offering micron-level detail of retinal and choroidal architecture. Spectral-domain systems provide axial resolutions close to 5 μm, enabling visualization of subtle microstructural alterations [39]. Enhanced Depth Imaging (EDI) and Swept-Source OCT (SS-OCT) further improve imaging depth and clarity, facilitating better assessment of deeper ocular layers such as the choroid and sclera [40].

Anterior Segment OCT (AS-OCT) adapts this technology for detailed imaging of the eye’s anterior structures, including the cornea, iris, anterior chamber, and lens [41,42]. It offers similarly fine axial resolution and benefits from broader scan ranges and deeper penetration with modern swept-source and spectral-domain platforms. In the setting of ocular melanoma, particularly involving the iris or anterior chamber angle, AS-OCT proves useful in delineating lesion margins, evaluating internal characteristics, and detecting angle involvement. This precise structural visualization supports clinical decision-making in both diagnosis and management of anterior segment tumors.

### 3.2. Posterior Segment Tumors: Choroidal Melanoma

OCT, particularly spectral-domain (SD-OCT) and enhanced depth imaging (EDI-OCT), has significantly advanced the characterization of choroidal melanoma by offering high-resolution cross-sectional views of the retina and choroid. It provides critical insight into tumor-associated changes that may not be visible on fundus examination or ultrasonography, thus playing a central role in differentiating benign from malignant pigmented fundus lesions [7,16,24].

One of the most frequently observed OCT features in choroidal melanoma is subretinal fluid (SRF), which is present in over 50% of cases and is considered a key indicator of tumor activity and potential for growth [4,10,14]. This fluid, often accumulating between the neurosensory retina and the RPE, may be associated with exudative retinal detachment and contributes significantly to visual symptoms. Some studies suggest that quantitative measures of SRF, such as the optical density ratio (ODR), could help distinguish melanomas from choroidal nevi, which typically do not produce SRF or cause overlying retinal disturbance [10].

Additional retinal changes detectable on OCT include RPE alterations—such as irregularity, hyperreflectivity, or focal detachment—and retinal thinning or atrophy, particularly overlying larger or more chronic lesions [1,11,13]. Hyper-reflective dots within or above the tumor surface are also common; these may represent lipofuscin-laden macrophages or degenerative retinal pigment granules, and their presence has been linked with increased risk of malignancy [1,7,28]. Occasionally, OCT reveals intraretinal cystoid spaces or evidence of bacillary layer detachment, especially in more exudative lesions [11,14].

**Figure 1:**
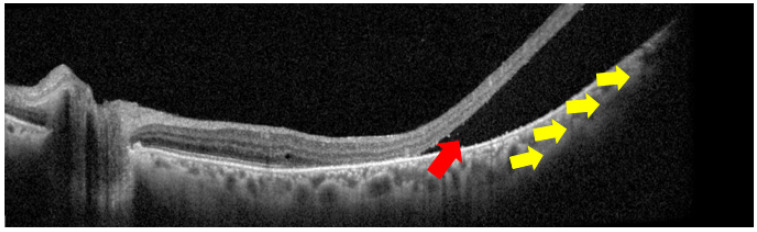
Optical coherence tomography scan in which two important signs of choroidal melanoma can be detected: exudative retinal detachment with subretinal fluid (red arrow) and choroidal profile elevation with mass effect (yellow arrows).

EDI-OCT and SS-OCT provide improved visualization of the choroid, including shallow elevations due to tumor mass effect and compression of choroidal vasculature, particularly in small or medium-sized melanomas [7,12,16]. These platforms can also detect dome-shaped or mushroom-like configurations, especially in cases where the tumor has breached Bruch’s membrane, which corresponds to more advanced lesions [1,30] [Figure 1].

Such findings may be critical for differentiating choroidal melanoma from central serous chorioretinopathy, hemorrhagic PEDs, or choroidal metastases, all of which may have overlapping features but lack the same constellation of signs [13,22,24].

Key datasets include Cennamo et al., a prospective feasibility series of 116 eyes focused on small posterior-pole tumors with 3 × 3 and 6 × 6 mm fields [43]; Güner et al., a single-center retrospective cohort of 236 melanomas that detailed size-stratified subretinal fluid and reported bacillary layer detachment [44]; and Zhang et al., a five-year swept-source OCT study of 305 eyes across ten entities that used multivariable modeling to identify features associated with malignancy [45]. Taken together—and alongside other prospective and retrospective reports—these studies converge on similar patterns, such as subretinal fluid and outer-retinal disruption as activity markers and SS-OCT features like overlying choriocapillaris loss, optical shadowing, and subretinal fluid extending beyond the lesion margin increasing post-test probability, while also underscoring methodological heterogeneity in device wavelength and field, segmentation, and case mix, and the need for harmonized, prospective validation.

Importantly, OCT is useful not only in initial diagnosis but also in monitoring the natural history or therapeutic response of choroidal melanomas. After radiotherapy, OCT can detect early signs of radiation retinopathy, including capillary dropout, macular edema, and retinal ischemia, even before visual acuity is affected [21,25]. Some case series have also demonstrated the value of serial OCT imaging in detecting subclinical tumor growth over time, particularly in small lesions initially presumed benign [15,27].

While OCT alone cannot penetrate the full tumor thickness as reliably as ultrasonography, it offers unparalleled resolution (~5 μm in SD-OCT) and is particularly helpful in detecting subtle retinal signs that may support a malignant diagnosis. Combined with clinical examination, ultrasonography, fluorescein angiography, and other modalities, OCT forms an indispensable component of the modern multimodal imaging approach to choroidal melanoma [16,19,26]. In addition, novel intraoperative techniques have expanded OCT’s utility beyond diagnosis. Microscope-integrated OCT (MIOCT), combined with real-time 4D-guided imaging, has been employed to facilitate 27-gauge transvitreal biopsy of choroidal melanomas. This approach enables precise sampling from targeted tumor depths while minimizing the risk of damaging the overlying retina or causing intrachoroidal hemorrhage. Despite its promise in improving biopsy accuracy, this technique is not widely adopted due to procedural risks and limited added clinical value, particularly when diagnosis is confidently achieved through non-invasive imaging alone [46,47].

### 3.3. Anterior Segment Tumors: Iris and Ciliary Body Melanoma

**Figure 2:**
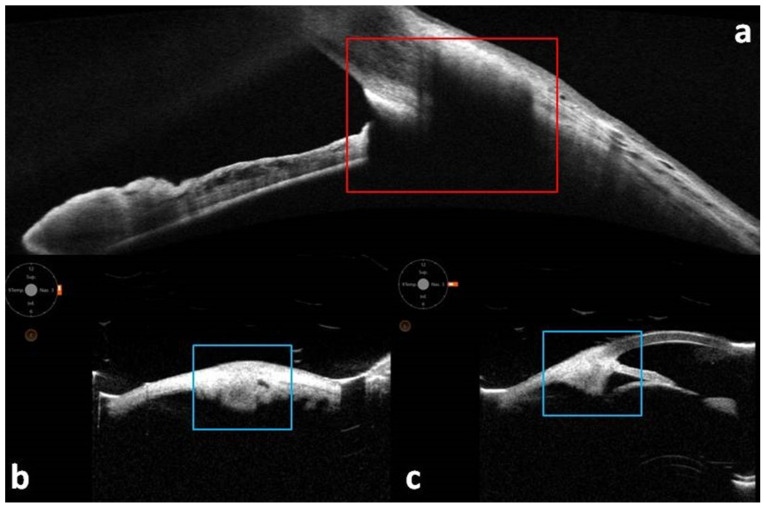
Multimodal imaging of the anterior segment in a patient with a ciliary body melanoma previously treated with brachytherapy. (**a**) Optical coherence tomography scan where it is not possible to evaluate the lesion, but it is only possible to see a widespread shadowing of the analyzed area (red square) related to the device resolution which does not allow such assessment. Conversely, the use of ultrasound biomicroscopy in transversal (**b**) and longitudinal (**c**) scan permits a clear visualization of the lesion, also allowing its measurement and evaluation over time (blue squares).

AS-OCT has emerged as a valuable tool in the evaluation of anterior uveal tumors, particularly those arising from the ciliary body and iris [48]. While Ultrasound Biomicroscopy (UBM) remains the gold standard for imaging structures posterior to the iris due to its superior tissue penetration, AS-OCT offers distinct advantages in terms of resolution, patient comfort, and ease of use. Its axial resolution of approximately 5–7 μm enables detailed visualization of anterior segment anatomy and allows detection of subtle structural alterations that may suggest an underlying mass, even when the lesion itself is not directly visible [49]. In contrast, high-frequency UBM (≈35–50 MHz) penetrates pigmented tissues and opaque media, directly visualizing the pars plicata/plana, posterior lesion margins, ciliary sulcus, and potential scleral/extrascleral extension—capabilities that make it the reference modality for posteriorly located or heavily pigmented lesions [49,50,51,52,53]. In practice, UBM is preferred when any of the following apply: lesion thickness > 2 mm or base > 2.5 mm; marked pigmentation with posterior shadowing; suspected ciliary body/pars plicata extension; limited corneal clarity, hyphema, or shallow chambers; or when precise posterior margin mapping is required for surgery or plaque planning [49,50,51,52,53]. This complementarity is illustrated in Figure 2: in a post-brachytherapy ciliary body melanoma, AS-OCT provides only nondiagnostic shadowing of the region of interest, whereas transverse and longitudinal UBM clearly delineate the mass, allow thickness/base measurements, and support reproducible serial monitoring.

In cases of ciliary body melanoma, AS-OCT can reveal early indirect signs such as iris bowing, anterior chamber shallowing, and angle narrowing. Although its penetration is more limited than UBM, modern swept-source and Fourier-domain platforms have significantly enhanced imaging depth and field of view [50,51]. In a retrospective analysis by Mirzayev et al. including data from 42 eyes of 38 patients with iris and ciliary body tumors, 14 were melanomas, 14 IPE cysts, 7 nevi, 3 Lisch nodules, 2 iris stromal cysts, 1 pars plana cyst, and 1 iris mammillation. The study demonstrated that anterior segment swept-source OCT (AS SS-OCT) offered equivalent visualization of anterior tumor margins compared to UBM, while the latter remained superior for evaluating posterior margins, especially in melanocytic tumors and IPE cysts. Bland–Altman analysis showed strong agreement between the two modalities for lesions smaller than 2.5 mm in base and 2 mm in thickness, supporting AS SS-OCT as a useful non-contact option for small, minimally elevated iris tumors [51]. However, as a single-center, retrospective series with a modest and heterogeneous lesion mix and device-specific acquisition parameters, the study is prone to spectrum and verification bias; UBM was used as the operational reference standard, histopathologic confirmation was not available, and masking of graders was not reported. The Bland–Altman agreement applied chiefly to small, minimally elevated lesions (base < 2.5 mm; thickness < 2 mm), so performance in thicker, more pigmented, or posteriorly extending tumors—particularly ciliary body lesions with posterior margins—remains uncertain. Intersession repeatability, inter-device agreement, and impact on management decisions were not assessed, which limits external validity and generalizability. In their systematic review of 41 studies including 545 iris and ciliary body lesions, Mirzayev et al. highlighted the utility of anterior segment OCT as a non-invasive imaging tool particularly effective in diagnosing iris and angle tumors, including pigment epithelial and stromal cysts, while noting its limitations in evaluating deeper ciliary body involvement [52].

In iris melanoma, AS-OCT provides high-resolution cross-sectional images that aid in assessing tumor thickness, basal diameter, and architectural disruption. These lesions typically appear as hyperreflective masses with posterior shadowing, and AS-OCT is especially effective in distinguishing amelanotic or superficially located tumors from benign nevi [53]. Its non-contact nature and high patient tolerability make it ideal for serial imaging. Combined with UBM and slit-lamp biomicroscopy, AS-OCT enhances the accuracy of diagnosis, supports risk stratification, and assists in long-term surveillance of anterior uveal tumors.

## 4. OCT Angiography

### 4.1. Principles and Capabilities

OCTA is a non-invasive imaging technique that enables high-resolution, depth-resolved visualization of the retinal and choroidal vasculature without the need for dye injection [54]. It operates by detecting motion contrast generated by moving erythrocytes within blood vessels, distinguishing flow from static tissue based on signal decorrelation across sequential OCT B-scans at the same retinal location. The resulting data is processed to produce en face angiograms of different vascular plexuses [55].

Modern OCTA systems segment the retinal and choroidal layers into distinct slabs—typically including the superficial and deep capillary plexuses, outer retina, and the choriocapillaris—allowing a three-dimensional assessment of vascular architecture [56]. This depth-resolved approach is particularly useful in uveal melanoma, where visualization of tumor-associated vascular changes in both the retina and choroid is essential. By offering detailed vascular maps with axial resolution as fine as ~5 μm and avoiding the risks of intravenous dyes, OCTA has emerged as a valuable adjunct to traditional angiographic techniques.

Importantly, recent advancements in swept-source OCTA (SS-OCTA), which uses longer wavelengths (~1050 nm) and faster scanning speeds, have improved choroidal imaging by enabling deeper penetration through pigmented tissues and dense lesions, which are common in uveal melanoma. These technological refinements support the use of OCTA for non-invasive vascular monitoring in both diagnostic and post-treatment settings [57,58].

### 4.2. Tumor Vasculature and Diagnostic Use

While OCTA is limited in its ability to penetrate thick or heavily pigmented choroidal melanomas due to signal attenuation and shadowing, it remains a valuable adjunct for assessing tumor-associated retinal and choroidal vascular changes [59]. In particular, OCTA has shown promise in identifying microvascular alterations that may not be visible with structural OCT or fundus examination alone.

One of its key diagnostic applications is the non-invasive evaluation of intrinsic tumor vasculature. Although deep signal loss is common in larger melanomas, OCTA can detect irregular and tortuous vascular networks at the lesion margins or within thinner tumors; when elevation or SRF distorts layers, careful manual segmentation is often required to avoid missing true tumor flow. Pellegrini et al.—observational case series, 22 melanomas—reported coexisting large vascular trunks and fine capillary-like networks within melanoma after manual correction, underscoring the importance of artifact control [60]. Building on this, Zhang et al.—single-center retrospective cohort, 102 consecutive cases with 87 analyzable scans, 61 melanomas and 26 nevi—introduced a five-grade choroidal-slab system capturing the density/morphology of disorganized networks; in that series, 98% of melanomas were Grade ≥ 2, whereas nevi split into either well-organized patterns (Grade 0–1) or “melanoma-like” disorganized networks (Grade 2–4), with high-risk nevi (by multimodal criteria) far more likely to display melanoma-like networks. Melanomas also carried a substantially higher choriocapillaris flow-deficit burden quantified within tumor-centered regions [61].

Additionally, the technique enables detection of secondary retinal changes, such as enlargement of the foveal avascular zone (FAZ), perifoveal capillary rarefaction, and signs of ischemia in tissues adjacent to the tumor [6].

Garcia-Arumi Fuste et al.—authors of a comparative cross-sectional study of consecutive patients, 36 eyes total with 18 nevi and 18 melanomas—found that melanomas more often showed imprecise margins, hyporeflective choriocapillaris, multiple avascular areas, and choroidal vascular abnormalities (thick networks/loops), while nevi tended to have well-delimited margins, hyperreflective choriocapillaris, and fewer avascular areas—features that support differential diagnosis in small lesions [62]. Consistent with paratumoral remodeling, Pellegrini et al. demonstrated that eyes with posterior uveal melanoma exhibited significant enlargement of the FAZ and reduced vessel density in both the superficial and deep retinal capillary plexuses compared to healthy fellow eyes, suggesting a paratumoral vascular remodeling process [63]. Similarly, Li et al. reported capillary dropout, flow voids, and a decrease in perifoveal perfusion in eyes harboring choroidal melanomas, with changes sometimes extending beyond the visible tumor margins [6]. These findings indicate that OCTA can detect early, subclinical microvascular compromise, which may have diagnostic and prognostic implications, particularly in patients undergoing surveillance or when planning treatment.

After treatment, OCTA metrics are clinically informative. Jung—prospective single-center cross-sectional study, 24 participants after proton beam therapy—found larger foveal avascular zone area and perimeter, reduced parafoveal and perifoveal superficial plexus density, and lower macular choriocapillaris flow in treated eyes versus fellow eyes. Best-corrected visual acuity correlated most strongly with foveal avascular zone size, deep-plexus density, and radiation dose to the fovea [64]. Torkashvand—retrospective observational case series, 31 eyes after ruthenium-106 plaque—showed that early subclinical injury concentrates in the deep capillary plexus with deep foveal avascular zone enlargement and decreased deep-plexus vessel and skeleton density even without clinical radiation maculopathy; higher foveal and optic-disk dose predicted a “burnout” vascular phenotype [65]. Binkley—longitudinal single-patient study over four years after iodine-125 plaque—mapped sector-wise macular capillary loss that tracked with higher plaque dose, while overt fundus markers waned, highlighting OCTA’s sensitivity to ongoing microvascular attrition [66]. Yang—prospective, nonrandomized interventional comparative study, 45 eyes after iodine-125 plaque, 15 received six scheduled conbercept injections and 30 served as untreated controls—showed higher superficial vessel density at six months with conbercept, relative stability versus decline in controls, and attenuation of foveal avascular zone enlargement, but no sustained advantage by nine to twelve months, suggesting a time-limited protective effect that needs randomized confirmation [67].

Taken together across plaque brachytherapy series, OCTA acts as an early, quantitative biomarker of radiation maculopathy and treatment response, enabling dose-aware surveillance and timely anti-VEGF intervention; standardized fields, layer definitions, and metrics should be built into routine post-brachytherapy follow-up.

Thus, although OCTA is not a primary imaging modality for direct tumor visualization in uveal melanoma, it plays a valuable role in evaluating secondary vascular alterations and supporting diagnostic decision-making in a multimodal imaging context.

### 4.3. Strengths and Limitations

OCTA offers several compelling advantages in the evaluation and follow-up of patients with uveal melanoma. As a non-invasive and dye-free modality, it eliminates the risks associated with fluorescein or indocyanine green angiography, making it safer and more tolerable for patients—particularly for repeated follow-ups [68]. OCTA also enables layer-by-layer visualization of the retinal and choroidal microvasculature, facilitating detailed assessment of vascular alterations such as capillary dropout, FAZ changes, and microaneurysms, which may indicate early radiation damage or tumor-associated ischemia [69].

In addition to its qualitative insights, OCTA provides quantitative metrics, including vessel density and perfusion indices, allowing for objective monitoring of disease progression and treatment response. Its rapid acquisition and depth-resolved imaging make it a valuable adjunct in ocular oncology, particularly for macular evaluation in patients undergoing radiotherapy [69].

However, OCTA also presents limitations:

- **Penetration and shadowing:** Melanin and thickness attenuate signal and can obscure deep choroid or the tumor core, so an apparent absence of vasculature may reflect physics rather than biology.
- **Segmentation and projection artifacts:** Elevation, subretinal fluid, and remodeling challenge automated slabs; manual Bruch’s membrane delineation and projection-artifact suppression are often required to avoid false findings.
- **Device and protocol heterogeneity:** Platforms, wavelengths, scan sizes, and algorithms differ, which limits direct comparison and pooled thresholds; the Zhang grading requires external, device-agnostic validation [61].
- **Selection and spectrum bias:** Single-center series often over-represent posterior, smaller, well-fixating lesions; peripheral or thick tumors and poor-quality images are frequently excluded, which can inflate reported yields.
- **Biological attribution:** OCTA measures motion contrast; reduced decorrelation may indicate slow flow rather than vessel loss, and anti-VEGF therapy can modify edema and secondarily influence metrics.
- **Incremental value and outcomes:** While Garcia-Arumi Fuste and Zhang show discriminative patterns [61,62], the added predictive value of OCTA over established multimodal risk models and ultrasound thickness still needs prospective evaluation with clinical endpoints and, where feasible, genetic or histologic anchors.
- **Field of view and topology:** Standard 3–12 mm fields may miss peripheral margins or extratumoral choroidopathy; dose-mapped sector analysis is promising but remains labor-intensive and not widely available [70,71].

To operationalize these caveats, Table 1 summarizes common OCT and OCTA pitfalls in uveal melanoma and the practical steps that reduce error at acquisition, processing, and interpretation.

Non-visualization of intrinsic tumor vasculature or choriocapillaris flow on OCTA should not be taken as absence of tumor vascularity or ischemia, particularly in thick or heavily pigmented lesions. Any OCT or OCTA finding that would change staging, margins, or treatment should be corroborated within a standardized multimodal pathway that includes ultrasonography and, when indicated, angiography. Nevertheless. despite these limitations, ongoing advancements in swept-source OCTA and enhanced algorithms for artifact correction continue to improve its diagnostic utility. When interpreted in conjunction with structural OCT and other imaging modalities, OCTA significantly enhances the clinician’s ability to non-invasively assess tumor-related and post-radiation microvascular changes.

## 5. Differential Diagnosis: Melanoma vs. Pseudomelanoma

The differentiation between uveal melanoma and pseudomelanomas is a keystone of accurate ocular oncology practice, as misdiagnosis may lead to unnecessary interventions or delayed treatment. Multimodal imaging, particularly OCT and OCTA, offers critical structural and vascular insights that can refine diagnostic accuracy.

Choroidal nevi are the most frequent mimickers of melanoma. While they may share pigmentation and location with melanomas, they tend to be smaller, flat or minimally elevated, and less likely to show associated retinal alterations. OCT often reveals an intact overlying retinal architecture, absence of SRF, and preserved RPE-Bruch’s membrane complex. In contrast, melanomas typically show dome-shaped elevation, RPE detachment or rupture, choroidal compression, and frequently associated subretinal fluid, even in small lesions [72,73].

Central serous chorioretinopathy (CSCR) can resemble amelanotic melanoma, particularly in chronic cases where RPE changes are prominent. However, the presence of serous retinal detachment without mass effect, a thickened choroid, and absence of intrinsic tumor vasculature on OCTA can help distinguish CSCR from true malignancy [74].

Choroidal hemangiomas, although elevated, typically have a homogenous internal reflectivity on B-scan ultrasound and lack the nodular or mushroom-shaped contour seen in melanomas. OCT may show serous retinal detachment, but the underlying lesion is often smoother, with less RPE disturbance. On OCTA, hemangiomas may show a coarse but organized vascular network, differing from the irregular or dropout patterns in melanomas [75,76].

Metastases are usually creamy yellow lesions, often associated with rapid onset visual symptoms. OCT typically shows shallow elevation with a “lumpy” anterior contour, minimal overlying SRF, and can be multifocal. OCTA findings are variable but may show choroidal vascular attenuation without the disorganized tumor vasculature seen in melanoma [77].

Pigmented epiretinal membranes may mimic superficial pigmented lesions. However, their position above the retina and lack of choroidal involvement can be easily confirmed with OCT.

The distinguishing multimodal imaging features of choroidal melanoma compared to its main clinical mimickers, including choroidal nevus, central serous chorioretinopathy (CSCR), choroidal hemangioma, and metastasis, highlighting differences in elevation, subretinal fluid presence, retinal pigment epithelium alterations, and vascular patterns on OCT and OCTA are given in Table 2.

This comparative table summarizes key features of choroidal melanoma, choroidal nevus, central serous chorioretinopathy (CSCR), circumscribed choroidal hemangioma, and choroidal metastasis based on elevation pattern, presence of subretinal fluid (SRF), retinal pigment epithelium (RPE) disruption, choroidal compression, OCT angiography (OCTA) vascular pattern, foveal avascular zone (FAZ) changes, growth behavior, and pigmentation. These parameters aid in differentiating malignant from benign or inflammatory lesions during noninvasive imaging evaluation.

## 6. Innovations and Future Perspectives

### 6.1. AI and Machine Learning Integration

The application of machine learning (ML) and artificial intelligence (AI) in ophthalmic imaging is rapidly evolving, offering substantial promise in the management of UM. These technologies are being designed to assist clinicians by automating the interpretation of complex OCT and OCTA datasets, enhancing diagnostic precision, and facilitating early detection of treatment-related complications [78].

In the diagnostic phase, ML models—particularly those using deep learning architectures such as convolutional neural networks (CNNs)—have shown capability in differentiating between malignant and benign intraocular lesions. By training algorithms on large annotated image datasets, these systems can learn to identify key features such as subretinal fluid, retinal pigment epithelium (RPE) alterations, tumor elevation patterns, and early vascular changes that are often indicative of UM. When applied to OCTA images, machine learning can also quantify capillary density, detect FAZ enlargement, and identify perfusion abnormalities that may otherwise go unnoticed in manual assessment [79].

A significant advantage of machine learning in this context lies in its potential to support longitudinal monitoring. Algorithms can be developed to analyze changes in OCT/OCTA metrics over time—such as tumor-induced retinal thickening, macular edema progression, or post-radiation vessel dropout—thus enabling timely identification of progression or therapeutic response. This is particularly relevant in patients undergoing radiotherapy, where early detection of radiation maculopathy or ischemia is critical to prevent irreversible visual decline.

Moreover, AI can contribute to risk stratification by integrating imaging features with clinical variables (e.g., age, tumor location, treatment type), potentially enabling personalized monitoring protocols. Early experimental frameworks have demonstrated that machine learning models can predict visual outcomes or the likelihood of complications post-treatment based on baseline imaging features alone.

Deep learning on color fundus photography already shows real-world utility for front-end triage: Hoffmann and colleagues trained a ResNet-based classifier on 762 ultra-widefield and standard images to separate nevi, untreated melanoma, and irradiated melanoma with high accuracy, particularly for the irradiated class, suggesting AI can screen before confirmatory OCT/OCTA and ultrasound [80]. Sabazade’s group, using 802 photographs from 688 patients, built a two-stage U-Net that matched ocular oncologists for sensitivity in small melanomas while maintaining competitive specificity, reinforcing the role of AI as a safety-first triage tool [81]. Moving toward foundation models, Jackson et al. fine-tuned a large self-supervised retinal model on 27,181 images from 4255 patients and achieved strong melanoma–nevus discrimination, indicating that pretrained backbones can transfer well to ocular oncology and could be extended with OCT/OCTA channels [82]. In parallel, clinical-variable models without imaging can aid decisions at the slit lamp: Zabor et al. developed a LASSO model in 123 patients, externally validated in 240 additional cases, that accurately distinguished small melanoma from nevus using variables such as subretinal fluid, height, orange pigment, and disk distance, and delivered a bedside risk calculator [83]. For multimodal risk prediction, Tailor et al. assembled a multicenter cohort of 2870 nevi and showed that gradient-boosting models fusing fundus, OCT/FAF, and ultrasound generalize well on external data, with tumor thickness, largest basal diameter, distance to the nerve, and subretinal fluid ranking highest—features OCT/OCTA can augment with quantitative vascular metrics [84]. Finally, for automation of OCT workflows, Valmaggia et al. trained a 3D nnU-Net on 121 swept-source OCT volumes from 71 individuals to segment pigmented choroidal lesions with high overlap and sub-millimeter boundary error, paving the way for reproducible volumetry and longitudinal change detection [85]. Together, these strands point to a practical pipeline: AI-assisted triage on fundus images, OCT/OCTA-guided confirmation and quantification, and multimodal prognostic models that integrate vascular metrics for surveillance and therapy planning [86].

Despite these advances, several barriers hinder widespread clinical integration. The lack of standardized imaging protocols across devices and institutions complicates the training and validation of robust, generalizable models. Additionally, most studies to date have been limited by small sample sizes or single-center data, raising concerns about model overfitting and limited external applicability. Regulatory, ethical, and data governance issues—particularly concerning patient privacy and explainability of AI decisions—also require careful consideration. Implementation should follow practical ethics and value principles—model transparency with clinician oversight, routine bias auditing across diverse populations, consent-respectful data use, and prospective evidence that OCT/OCTA-AI reduces avoidable referrals or vision loss at an acceptable cost compared with current multimodal pathways.

Future research should aim at the development of multicenter OCT/OCTA image databases for UM and its mimickers, facilitating algorithm training with diverse real-world data. Incorporating federated learning frameworks may help overcome data-sharing limitations while preserving patient confidentiality. Clinical trials that assess AI-assisted decision-making tools prospectively will be essential to determine their actual impact on diagnostic accuracy, workflow efficiency, and patient outcomes.

In conclusion, the integration of machine learning into OCT and OCTA analysis represents a transformative direction in the imaging and management of UM. While technical and practical challenges remain, ongoing innovation and rigorous validation hold the potential to make AI-driven tools indispensable assets in ocular oncology.

### 6.2. Multimodal Imaging Protocols

The integration of multiple imaging modalities is increasingly recognized as essential for the accurate diagnosis, monitoring, and management of UM. While OCT and OCTA provide high-resolution structural and vascular detail, they each have inherent limitations—such as poor penetration through heavily pigmented or thick lesions and difficulty in assessing deep extrascleral extension. As such, combining these techniques with complementary tools such as fundus autofluorescence (FAF), B-scan ultrasonography, and even fluorescein or indocyanine green angiography enhances diagnostic precision [3].

**Figure 3:**
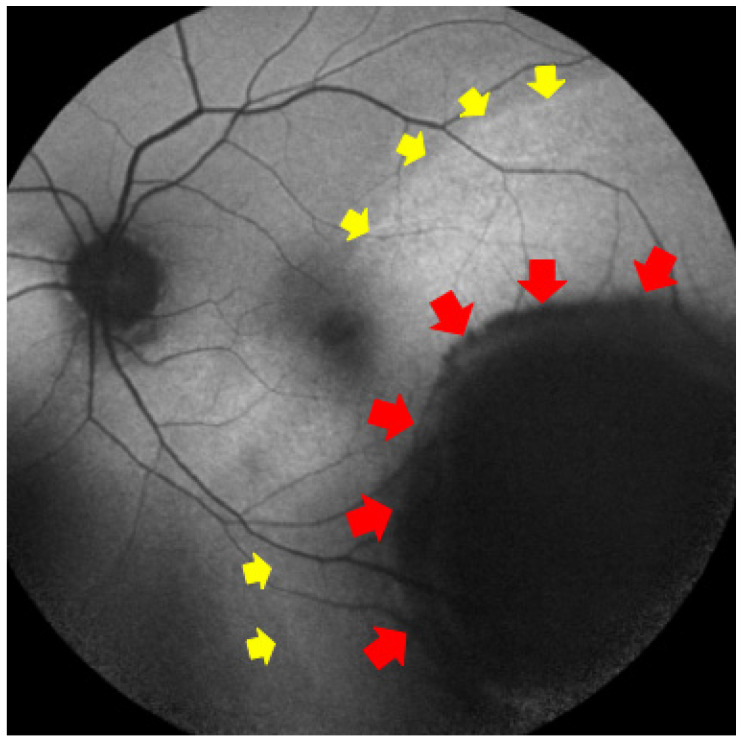
Fundus autofluorescence scan in which can be detected the pigmented lesion as hypoautofluorescence region (red arrows), with surrounding exudative retinal detachment (yellow arrows), that is considered as a malignant sign.

FAF plays a pivotal role in identifying lipofuscin deposition, a hallmark of malignant transformation, which may appear as hyperautofluorescent spots overlying choroidal melanomas. When interpreted alongside OCT findings such as subretinal fluid or RPE irregularities, FAF can help differentiate melanomas from benign lesions such as nevi or congenital hypertrophy of the retinal pigment epithelium (CHRPE) [87,88] [Figure 3].

Ultrasonography, particularly B-scan and standardized A-scan, remains the gold standard for assessing tumor thickness, internal reflectivity, and extrascleral extension—features not fully captured by OCT or OCTA [87,88,89].

Combining these modalities enables a layered diagnostic approach: OCT evaluates retinal and RPE architecture, OCTA maps intrinsic tumor vasculature and surrounding ischemia, FAF highlights metabolic changes at the RPE level, and ultrasonography confirms lesion dimensions and internal characteristics. This multimodal strategy reduces the risk of misdiagnosis, especially in ambiguous cases or when OCT signal attenuation limits visualization of posterior tumor margins. In support of multimodal risk assessment, Shields and colleagues analyzed 3806 choroidal nevi (2355 with follow-up) and introduced the TFSOM-DIM rubric—Thickness > 2 mm (US), subretinal Fluid (OCT), Symptoms ≤ 20/50, Orange pigment (autofluorescence), Melanoma hollowness (US), and tumor DIaMeter > 5 mm—demonstrating a stepwise increase in 5-year transformation risk from ~1% with no factors to ≥34–55% with three to five factors [90].

Recent studies have demonstrated that multimodal imaging not only improves baseline characterization but also facilitates longitudinal monitoring of treatment response and detection of complications such as radiation-induced maculopathy or tumor recurrence [64,65,66,67,87,91]. Moreover, standardized imaging protocols incorporating these modalities could improve inter-center diagnostic consistency, support the development of AI training datasets, and enhance clinical decision-making.

In summary, multimodal imaging represents a synergistic approach in ocular oncology. The combined use of OCT, OCTA, FAF, and ultrasonography leverages the strengths of each modality, offering a comprehensive and more reliable assessment of UM across its clinical spectrum.

### 6.3. Prognostic Imaging Biomarkers

The identification and validation of imaging biomarkers capable of predicting tumor behavior and treatment outcomes remain key challenges in the management of uveal melanoma. OCT) and OCTA offer the potential to non-invasively track changes in retinal and choroidal architecture and vasculature over time, which may serve as early indicators of disease progression or therapeutic response.

Among the most promising biomarkers is SRF, whose presence has been consistently associated with active tumor growth. Resolution or persistence of SRF following treatment—particularly plaque brachytherapy or proton beam therapy—may provide early insights into treatment efficacy. Similarly, RPE disruption, cystoid macular edema, and retinal thinning detected by OCT have been linked to poorer visual outcomes and may serve as surrogate endpoints in follow-up evaluations.

On the vascular side, OCTA-derived metrics such as enlargement of the FAZ, capillary dropout, and decreased vessel density in the perifoveal or peritumoral regions are gaining interest as potential prognostic indicators. Longitudinal studies are needed to determine whether these microvascular alterations correlate with clinical endpoints such as local recurrence, radiation retinopathy, or metastasis. To make biomarker use explicit and reproducible, we summarized in Table 3 the key OCT and OCTA metrics—covering SRF, CMT, FAZ and parafoveal densities in SCP and DCP, FD-300, RPC and RNFL measures, choriocapillaris flow indices, intratumoral vasculature grading, CC avascular patches, macular VSD, and BALAD—with risk directionality, primary clinical use, and the most significant representative evidence.

Despite promising early findings, few of these biomarkers have been prospectively validated or integrated into routine clinical risk stratification. Future research should focus on establishing normative databases, standardizing image acquisition and quantification methods, and correlating imaging features with histopathologic or molecular tumor profiles. Multicenter collaborations and prospective registries will be critical to assess the predictive value of these parameters across diverse populations and treatment modalities.

Ultimately, incorporating imaging biomarkers into prognostic models could improve personalized surveillance schedules, guide therapeutic decision-making, and serve as non-invasive surrogates in clinical trials evaluating novel treatments for UM.

## 7. Conclusions

OCT and OCTA have become essential components of the diagnostic and monitoring toolkit in UM. These modalities offer high-resolution, non-invasive visualization of anatomical and vascular changes, contributing to more accurate diagnosis, risk stratification, and surveillance of treatment response. While current limitations include reduced penetration in thick or heavily pigmented tumors, OCT and OCTA effectively complement traditional imaging methods such as ultrasonography and fluorescein angiography. Nevertheless, the present evidence base is dominated by single-center, retrospective studies and heterogeneous acquisition/segmentation protocols, which constrain generalizability and comparability, so pooled sensitivity/specificity and reliability estimates are not feasible; consequently, there is a pressing need for prospective, multicenter investigations with harmonized OCT/OCTA protocols, standardized metrics, and predefined clinical endpoints to validate quantitative biomarkers with explicit accuracy (ROC) and reproducibility (test–retest, inter-grader, inter-device) metrics, establish actionable thresholds, and confirm added value in routine care. Looking ahead, the integration of artificial intelligence, quantitative biomarkers, and multimodal imaging protocols holds great promise for advancing personalized care and improving clinical outcomes in patients with UM.

## Figures and Tables

**Table 1:** Common OCT and OCTA limitations in uveal melanoma with practical mitigations. Abbreviations: EDI enhanced depth imaging, FA fluorescein angiography, ICG indocyanine green angiography.

Limitation	Practical Mitigations
Penetration and shadowing from melanin or tumor thickness	Use swept-source or EDI settings and longer wavelength; increase averaging and sensitivity; scan from multiple angles; expand field with montage; always cross-check with structural B-scans and ultrasound before calling “no flow.”
Segmentation and projection artifacts	Manually re-draw Bruch’s membrane and adjust slabs; use projection-resolved processing; review en face maps alongside flow-overlaid B-scans; document any manual edits; avoid interpreting vessels that mirror superficial patterns.
Device and protocol heterogeneity	Standardize and report device, scan size, slab definitions, and QC thresholds; use consistent metrics (e.g., same vessel density masks); where possible, validate findings across platforms or analyze with device-agnostic measures.
Selection and spectrum bias	Enroll consecutive cases and report acquisition failures; intentionally include peripheral or thicker tumors and poorer fixation; use wide-field or montaged OCTA to capture off-posterior-pole disease; pre-specify inclusion/exclusion criteria.
Biological attribution of “no flow” vs. slow flow; treatment effects	Repeat scans to confirm; interpret decorrelation loss cautiously; time OCTA relative to anti-VEGF or corticosteroid therapy and record injection dates; corroborate with FA/ICG when management could change.
Unclear incremental value for prediction and management	Embed OCTA in prospective, multicenter studies; predefine endpoints (growth, radiation maculopathy, vision); test additive value over ultrasound and multimodal risk models with decision-curve analysis; anchor subsets with genetic or histologic data.
Limited field of view and topology	Use larger scans (9–12 mm) and montage; apply eye-tracking and fixation targets; for treated eyes, consider sector-based analysis aligned to radiation dose maps; add targeted peripheral scans when margins or extratumoral changes are suspected.

**Table 2:** Multimodal imaging features distinguishing choroidal melanoma from its clinical mimickers.

Feature	Choroidal Melanoma	Choroidal Nevus	CSCR	Choroidal Hemangioma	Metastasis
**Elevation**	Dome- or mushroom-shaped mass	Flat or minimally elevated	No true mass, serous detachment only	Smooth, dome-shaped elevation	Shallow, “lumpy” contour
**SRF Presence**	Common (>50% cases)	Rare	Typical feature	Frequent, localized	Occasional
**RPE Disruption**	Frequent (detachment, rupture)	Absent or minimal	RPE irregularities in chronic form	Minimal or absent	Variable
**Choroidal Compression**	Present	Absent	Absent	Absent	Rare
**OCTA Vascular Pattern**	Irregular, intrinsic flow or dropout	Absent or normal flow	Normal or dilated choroidal vessels	Coarse but organized vascular network	Attenuated or distorted vasculature
**FAZ Changes on OCTA**	Enlarged or irregular	Normal	Normal	May be slightly enlarged	Occasionally altered
**Growth Over Time**	Progressive	Stable	Transient/self-resolving	Usually stable	Rapid progression possible
**Pigmentation**	Variable, often pigmented	Typically pigmented	Usually non-pigmented	Orange-red or amelanotic	Amelanotic or yellowish

SRF: subretinal fluid; RPE: retinal pigment epithelium; OCTA: optical coherence tomography angiography; FAZ: foveal avascular zone; CSCR: central serous chorioretinopathy.

**Table 3:** Candidate OCT/OCTA biomarkers in uveal melanoma—layer or device, risk directionality, primary use, and validation. Risk directionality indicates whether higher or lower values are associated with greater malignancy risk or worse function. The validation column summarizes study design and representative sample sizes to clarify evidence strength. Abbreviations: OCT, optical coherence tomography; SS-OCT, swept-source OCT; OCTA, OCT angiography; PBRT, proton beam radiotherapy; FAZ, foveal avascular zone; SCP, superficial capillary plexus; DCP, deep capillary plexus; FD-300, perifoveal vessel density within a 300-µm ring; RNFL, retinal nerve fiber layer; RPC, radial peripapillary capillaries; CC, choriocapillaris; VSD, vessel skeleton density; BALAD, bacillary layer detachment.

Biomarker	Layer/Device & Scan	Risk Directionality (Higher vs. Lower)	Primary Use	Validation Status & Representative Evidence
Subretinal fluid (SRF) presence/extent	Structural OCT (SD/SS-OCT), macular and lesion B-scans	Presence/greater extent → higher malignancy risk and activity	Diagnosis, risk stratification, monitoring	Large multicenter risk model using multimodal criteria; part of established nevus → melanoma risk scores (Shields; validated on 3806 nevi; retrospective, multicenter) [90].
Central macular thickness (CMT)	Structural OCT, macular cube (6 × 6 mm)	Higher CMT (edema) → worse function post-radiation; not uniformly dose-dependent	Post-treatment monitoring	Torkashvand et al., retrospective single-center Ru-106, n = 31: thicker CMT and enlarged FAZ vs. fellow eyes; functional association variable [65]. Jung et al., prospective single-center PBRT, n = 24: mixed structure–function correlation across timepoints [64].
FAZ area (superficial plexus)	OCTA SCP, 3 × 3 or 6 × 6 mm	Larger FAZ → macular ischemia and worse BCVA	Post-radiation monitoring, prognosis	Jung et al., prospective single-center PBRT, n = 24: SCP/DCP FAZ enlargement tracks BCVA loss [64]. Shields et al., OCTA after plaque, n = 65: FAZ enlargement and parafoveal rarefaction in irradiated eyes [69].
FAZ area (deep plexus)	OCTA DCP, 3 × 3 or 6 × 6 mm	Larger deep FAZ → stronger association with BCVA loss than SCP	Post-radiation monitoring, prognosis	Torkashvand, retrospective single-center, 31 eyes (deep FAZ more critical biomarker) [65]; Jung, prospective single-center, 24 eyes (deep plexus metrics correlate with BCVA) [64].
Parafoveal vessel density (SCP)	OCTA SCP, parafovea 1–3 mm ring	Lower density → macular ischemia; weaker BCVA link than DCP	Post-radiation monitoring	Jung, prospective single-center, 24 eyes (SCP decrease vs. fellow eyes; BCVA correlation weaker) [64].
Parafoveal vessel density (DCP)	OCTA DCP, parafovea 1–3 mm ring	Lower density → stronger link to vision loss	Post-radiation monitoring, prognosis	Jung, prospective single-center, 24 eyes (parafoveal/perifoveal DCP ↓ correlates with BCVA) [64]; Torkashvand, retrospective single-center, 31 eyes (earliest subclinical change at DCP) [65].
FD-300 (perifoveal density in 300-µm ring)	OCTA derived (around FAZ), typically 3 × 3 mm	Lower FD-300 → macular ischemia	Baseline assessment, monitoring	Yang et al., prospective nonrandomized interventional after I-125, n = 45 (15 conbercept/30 control): FD-300 lower in UM vs. fellow at baseline; early stabilization at 6 mo with anti-VEGF, waning by 9–12 mo [67]. Li et al., treatment-naïve UM vs. fellow eyes: parafoveal microvasculature altered on OCTA [6].
Radial peripapillary capillary (RPC) density and RNFL thickness	OCTA ONH RPC slab; OCT RNFL	Lower RPC and thinner RNFL → radiation papillopathy burden	Post-radiation monitoring	Jung et al., prospective PBRT, n = 24: RPC and RNFL thinning in treated eyes with limited direct BCVA linkage [64]; aligns with broader radiation retinopathy literature [12].
Choriocapillaris flow metrics (flow ratio/deficits)	OCTA choriocapillaris slab, 3 × 3 to 6 × 6 mm	More flow voids/lower flow ratio → choroidal ischemia; diagnostic adjunct	Diagnosis adjunct, post-radiation monitoring	Torkashvand et al., retrospective, n = 31: increased CC flow deficits after Ru-106 [65]. Jung et al., prospective, n = 24: CC flow ratio declines post-PBRT [64]. Additional SS-OCT/OCTA series describe CC shadow mitigation and tumor-adjacent changes (Pellegrini n = 22; Greig small cohort) [57,60,70].
Intrinsic tumor vasculature grade (disorganized loops/networks)	SS-OCTA choroid slab; 6–12 mm; grading 0–4	Higher grade (2–4) → melanoma vs. nevus; flags high-risk nevi	Differential diagnosis, risk stratification	Zhang et al., consecutive SS-OCTA cohort, n = 102: higher intralesional disorganization in melanomas; device-agnostic grading proposed; external validation pending [61]. Greig et al., small SS-OCTA series: qualitative differences between nevi and small melanomas [57].
Patchy avascular areas at choriocapillaris over lesion	OCTA choriocapillaris slab	Presence/greater extent → melanoma over nevus	Differential diagnosis	Zhang et al., n = 102: lesion-overlying CC avascular patches more frequent in melanoma [61]; corroborated by smaller SS-OCTA case series [57,60].
Vessel skeleton density (VSD) (macula)	OCTA post-processing metric (3 × 3 or 6 × 6 mm)	Lower VSD → capillary dropout; tracks sector dose	Longitudinal monitoring, dose–response mapping	Binkley et al., longitudinal single-patient case, 4-year follow-up after I-125: sector-wise VSD loss tracks higher plaque dose; requires cohort-level validation [66].
Bacillary layer detachment (BALAD)	Structural OCT through lesion apex	Presence → exudative activity; larger tumors	Disease activity marker	Güner et al., retrospective melanoma cohorts: BALAD associated with tumor-related SRF and exudation; prospective UM validation still needed [44].

## Data Availability

Data sharing is not applicable to this article as no new data were created or analyzed in this study.
